# Deep learning regressor model based on nigrosome MRI in Parkinson syndrome effectively predicts striatal dopamine transporter-SPECT uptake

**DOI:** 10.1007/s00234-023-03168-z

**Published:** 2023-05-20

**Authors:** Yun Jung Bae, Byung Se Choi, Jong-Min Kim, Walid Abdullah AI, Ildong Yun, Yoo Sung Song, Yoonho Nam, Se Jin Cho, Jae Hyoung Kim

**Affiliations:** 1 Departments of Radiology, Seoul National University Bundang Hospital, Seoul National University College of Medicine, Seongnam, Republic of Korea; 2 Departments of Neurology, Seoul National University Bundang Hospital, Seoul National University College of Medicine, 82, Gumi-ro 173beon-gil, Bundang-gu, 13620 Seongnam, Republic of Korea; 3 Division of Computer Engineering, Hankuk University of Foreign Studies, Yongin, Republic of Korea; 4 Departments of Nuclear Medicine, Seoul National University Bundang Hospital, Seoul National University College of Medicine, Seongnam, Republic of Korea

**Keywords:** Magnetic resonance imaging, Parkinsonism, Susceptibility-weighted imaging, Dopamine transporter imaging

## Abstract

**Purpose:**

Nigrosome imaging using susceptibility-weighted imaging (SWI) and dopamine transporter imaging using ^123^I-2β-carbomethoxy-3β-(4-iodophenyl)-N-(3-fluoropropyl)-nortropane (^123^I-FP-CIT) single-photon emission computerized tomography (SPECT) can evaluate Parkinsonism. Nigral hyperintensity from nigrosome-1 and striatal dopamine transporter uptake are reduced in Parkinsonism; however, quantification is only possible with SPECT. Here, we aimed to develop a deep-learning-based regressor model that can predict striatal ^123^I-FP-CIT uptake on nigrosome magnetic resonance imaging (MRI) as a biomarker for Parkinsonism.

**Methods:**

Between February 2017 and December 2018, participants who underwent 3 T brain MRI including SWI and ^123^I-FP-CIT SPECT based on suspected Parkinsonism were included. Two neuroradiologists evaluated the nigral hyperintensity and annotated the centroids of nigrosome-1 structures. We used a convolutional neural network-based regression model to predict striatal specific binding ratios (SBRs) measured via SPECT using the cropped nigrosome images. The correlation between measured and predicted SBRs was evaluated.

**Results:**

We included 367 participants (203 women (55.3%); age, 69.0 ± 9.2 [range, 39–88] years). Random data from 293 participants (80%) were used for training. In the test set (74 participants [20%]), the measured and predicted ^123^I-FP-CIT SBRs were significantly lower with the loss of nigral hyperintensity (2.31 ± 0.85 vs. 2.44 ± 0.90) than with intact nigral hyperintensity (4.16 ± 1.24 vs. 4.21 ± 1.35, *P* < 0.01). The sorted measured ^123^I-FP-CIT SBRs and the corresponding predicted values were significantly and positively correlated (ρ_c_ = 0.7443; 95% confidence interval, 0.6216–0.8314; *P* < 0.01).

**Conclusion:**

A deep learning-based regressor model effectively predicted striatal ^123^I-FP-CIT SBRs based on nigrosome MRI with high correlation using manually-measured values, enabling nigrosome MRI as a biomarker for nigrostriatal dopaminergic degeneration in Parkinsonism.

**Supplementary information:**

The online version contains supplementary material available at 10.1007/s00234-023-03168-z.

## Introduction

The main pathologic process underlying Parkinsonism, including Parkinson’s disease and multiple system atrophy, is α-synuclein-mediated nigrostriatal dopaminergic cell loss and iron overload in the substantia nigra (SN) of the midbrain [1]. The dopaminergic cell loss in the SN occurs in structures called nigrosomes, which are three-dimensional (3D) organizations of calbindin-poor zones within calbindin-rich neuropils of the nigral complex [2, 3].

The recent development of high-resolution magnetic resonance imaging (MRI), primarily susceptibility-weighted imaging (SWI), has enabled the direct visualization of the nigral structures in the SN [4–11]. The normal nigrosome appearance, showing hyperintensity between the hypointense areas of the SN, can be lost in idiopathic Parkinson’s disease, multiple system atrophy, and progressive supranuclear palsy, and even in premotor disease, such as isolated rapid-eye-movement sleep behavior disorder, which can support the clinical diagnosis of the diseases [4–11]. However, although Parkinsonism is a “progressive” neurodegenerative disease, the detection of nigral hyperintensity on SWI is a matter of “presence or absence,” which precludes the quantification of the nigrosome status, limiting the ability of SWI to monitor disease severity or progression. Contrarily, dopamine transporter (DaT) imaging, such as ^123^I-2β-carbomethoxy-3β-(4-iodophenyl)-N-(3-fluoropropyl)-nortropane single-photon emission computerized tomography (^123^I-FP-CIT SPECT), can provide information regarding nigrostriatal function based on quantified DaT uptake in the striatum; thus, it can diagnose Parkinsonism and monitor the disease [12, 13]. However, compared with MRI, interpretation of SPECT does not depend on absolute change but on comparing the counts between normal and abnormal regions; in addition, SPECT is associated with the risk of radiation exposure.

A few studies have focused on the agreement between SWI and ^123^I-FP-CIT SPECT [8, 14]. If the loss of nigral hyperintensity on SWI also progresses over time in correlation with the reduction in striatal DaT uptake on SPECT, we should be able to model the relationship between nigral imaging and SPECT to predict nigrostriatal dopaminergic degeneration on easily accessible MRI.

In this study, we aimed to develop a deep-learning-based regressor model that can predict striatal DaT uptake on nigrosome MRI, assessing the feasibility of deep learning for the quantitative interpretation of nigrosome imaging in the clinical setting for Parkinsonism. The purpose of this study was to correlate 3 T SWI with the manually measured ^123^I-FP-CIT in the continuous domain, instead of dividing the degree space into normal and abnormal subranges, to create MRI biomarkers for Parkinsonism correlated with nigrostriatal dopaminergic degeneration.

## Methods

This prospective case-control study was approved by the Institutional Review Board of our institution. It was conducted in accordance with the Helsinki Declaration. Written informed consent was obtained from all study participants.

### Study participants

Consecutive patients who visited the movement disorder center in our referral institution between February 2017 and December 2018 were eligible for the study. All were clinically assessed by a neurologist with 22 years of experience in movement disorders. Those who had suspected parkinsonian symptoms were enrolled if they underwent 3 T brain MRI examination including SWI and concurrent ^123^I-FP-CIT SPECT. Those who exhibited severe motion artifacts on MRI, especially SWI, and who demonstrated vascular ischemia in the striatum and/or occipital cortex on MRI, which could lead to false-positive results in the calculation of specific binding ratios (SBRs) on ^123^I-FP-CIT SPECT, were excluded. The clinical diagnosis of each participant was made in accordance with the established criteria as follows: idiopathic Parkinson’s disease [15], multiple system atrophy [16], progressive supranuclear palsy [17], isolated rapid-eye-movement sleep behavior disorder [18], drug-induced Parkinsonism [19], essential tremor [20], vascular pseudo-Parkinsonism [21], cerebellar ataxia [22], Fahr’s syndrome [23], and normal pressure hydrocephalus [24].

### MRI protocol

MRI was performed using a 3 T MRI scanner (Ingenia and Ingenia CX; Philips, Amsterdam, The Netherlands) with a 32-channel SENSE head coil. SWI was obtained using an imaging plane perpendicular to the midbrain. Imaging parameters for SWI were as follows: 3D multi-echo fast-field-echo sequence; repetition time, 88 ms; total echoes, 5; first echo time, 10 ms; echo interval, 10 ms; flip angle, 10°; field-of-view, 192 × 192 mm^2^; voxel size, 0.5 × 0.5 × 1 mm^2^; 30 slices; scan time, approximately 4 min. Based on SWI acquisition, susceptibility map-weighted imaging (SMWI) was generated from the multi-echo fast-field-echo complex images to improve the visibility of the nigrosome structures [25]. Post-processing was performed using MATLAB (MathWorks, Natick, MA, USA) according to a previously described method [26]. The first step was to generate magnitude images using multi-channel magnitude images. After correcting the phase offset of the individual channels, the phase images were combined as a complex mean. Next, the magnitude images at each of the five echoes were combined into a single image using the root sum of squares. Phase images at different echo times were unwrapped using the Laplacian algorithm, and the combined frequency was calculated for each voxel. Then, the background field was removed from the frequency images, and quantified susceptibility mapping was performed using the improved sparse linear equation and least-squares method [27]. A quantified susceptibility mapping mask was generated from the quantified susceptibility mapping for susceptibility contrast weighting. Finally, an SMWI was produced by multiplying the multi-echo combined magnitude images with the quantified susceptibility mapping mask. The reconstruction slice thickness of SMWI was set at 1 mm.

### ^123^I-FP-CIT SPECT protocol

The participants were orally administered 6 mL of Lugol's solution before the ^123^I-FP-CIT injection. Scans were performed 3 h after the intravenous injection of 185 MBq of ^123^I-FP-CIT (DATrace-123™, Samyoung Unitech, Seoul, Korea) with a triple-headed rotating gamma camera system (Trionix XLT; Trionix Research Laboratory, Inc., Twinsburg, OH, USA) with low-energy, ultra-resolution, parallel hole collimators. Scans were acquired in 40 steps spanning 120°, with 40 s per step, via the step-and-shoot method. Images were reconstructed using filtered back projection with a Butterworth filter (cutoff frequency, 0.4 cycle/cm; order, 13), and attenuation correction using Chang's method (coefficient of 0.12/cm). First, a nuclear medicine physician with 17 years of experience visually interpreted the ^123^I-FP-CIT SPECT results. The reader was blinded to the clinical data and the MRI findings at the time of interpretation. The decision regarding the SPECT results for the diagnosis was based on visual inspection. For the quantification analysis, images were analyzed with a dedicated software program (DATquant, Xeleris 3.1, GE Healthcare, Chicago, IL, USA). Volumes-of-interest were defined automatically with DATquant, regarding the striatum (caudate nucleus and putamen), caudate nucleus, putamen, and occipital cortex. The SBRs of the striatal regions were calculated as follows: (mean counts in striatal regions – mean counts in the occipital cortex) / (mean counts in the occipital cortex). The averaged value was used for further analysis.

### MRI analysis

Two board-certified neuroradiologists (with 11 and 21 years of experience, respectively) independently evaluated the bilateral nigral hyperintensities on SMWI followed by resolving discrepancies by consensus. Normal nigral hyperintensity was defined as a focal linear or oval hyperintense area in the dorsolateral aspect of the SN surrounded by hypointensity [8, 14, 26, 28].

### Deep regressor model

Two neuroradiologists annotated the centroids of the nigrosome-1 structures on SMWI in consensus to crop a region-of-interest (ROI) with a fixed voxel size of 50 × 50 × 20, which sufficiently covered the entire nigrosome-1 structure on each side. These crops were used for the deep-learning-based regression analysis.

**Fig. 1:**
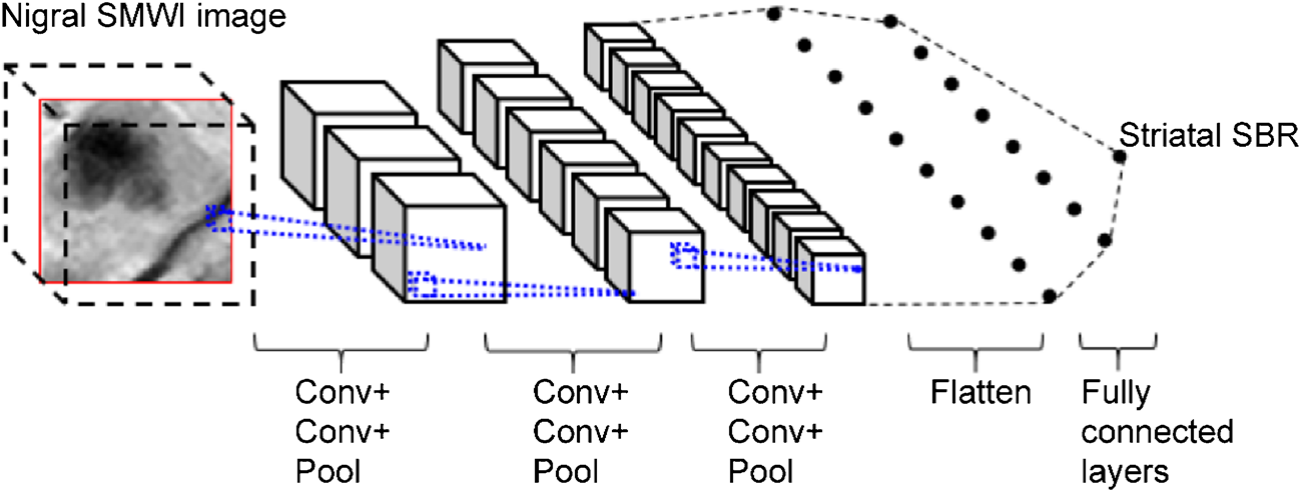
Deep regressor network. Three convolutional blocks were used, each with two convolutional layers (Conv) with a trailing max-pooling layer (Pool), to extract high-level feature maps. The feature map of the last convolutional block was flattened and passed through fully connected layers to predict a striatal SBR. SBR, specific binding ratio; SMWI, susceptibility map-weighted imaging

A convolutional neural network-based regression model was used to predict the target SBR because convolutional neural networks are widely used for learning discriminative image features. Specifically, we used the Visual Geometry Group (VGG)-style convolutional neural network (CNN) in our regression network (Fig. 1). Following the original order of the VGG layers, we used 3D convolutional blocks because the input nigrosome patch is a small 3D volume.

The network architecture followed a pattern similar to the VGG network where a stack of convolutional blocks first extracts high-level features from the input image, then a block of fully connected layers generates the final prediction from the extracted features. The configuration of the convolutional blocks (e.g., number and kernel size of the convolutional layers, type of the pooling layer) was also identical to the original VGG network. Nonetheless, the total number of convolutional blocks was reduced to three because of the small input size. Technical CNN architecture is described in Supplementary Materials.

The regressor was trained using the L2-loss as the objective function, the mean squared error between the model predictions and the corresponding true SBRs on SPECT. The training was aimed to minimize the mean squared error. A learning rate of 1e − 4 was used.

Though U-net [29] is a commonly used convolutional network for neuroimage analysis, U-net generates spatial output (e.g., segmentation) by adding a decoder block after the usual CNN encoder, which is not suitable for our task. In our method, our target SBR output is a single score assigned per patch, which is similar to the structure of the usual CNN output (e.g. class-probabilities, scalar values, etc.). Among the usual CNNs, VGG has been widely used in general medical image analysis [30]. Recent CNN architectures (e.g. ResNet [31]) have also been adopted in different medical image analysis tasks lately. However, VGG network with its simpler structure still remains a powerful tool under the small medical dataset. In our experiment, we also employed ResNet convolutional blocks for SBR prediction, which resulted in a poorer performance compared to the VGG blocks.

### Statistical analysis

Based on a single side, loss of nigral hyperintensity on SMWI was compared with the ipsilateral abnormal visual result on ^123^I-FP-CIT SPECT using the chi-square test, and the concordance rate was calculated. Next, striatal ^123^I-FP-CIT SBRs were compared according to the ipsilateral nigral hyperintensity status on SMWI. After training the deep regressor model, the predicted single-side striatal ^123^I-FP-CIT SBRs from the deep regressor model were also compared according to the status of nigral hyperintensity. The continuous predictions and ^123^I-FP-CIT SPECT SBRs were compared using residual analysis in terms of the absolute difference between the predicted and actual values. Normalized residuals were also computed after dividing the exact residuals by the SBR interval: the difference between the maximum and minimum values of the SBRs, as observed on ^123^I-FP-CIT SPECT. Lin’s correlation coefficient (ρ_c_) was computed to determine the agreement of the predicted SBRs with the actual SBRs on ^123^I-FP-CIT SPECT in the continuous domain. The 95% confidence interval (CI) of the correlation was computed using Fisher’s transform. Statistical significance was set at *P* < 0.05. Statistical analyses were performed using SPSS for Windows (version 25.0; IBM Corp., Armonk, NY, USA) and MedCalc for Windows (version 17.9; MedCalc Software, Ostend, Belgium).

## Results

### Clinical characteristics of the included participants

This study included 367 participants (sex, 203 women and 164 men; age, 69.0 ± 9.2 [range, 39–88] years). The clinical diagnoses of the participants included idiopathic Parkinson’s disease (*n =* 287), multiple system atrophy (*n =* 17), progressive supranuclear palsy (*n =* 14), isolated rapid-eye-movement sleep behavior disorder (*n =* 9), drug-induced Parkinsonism (*n =* 3), essential tremor (*n =* 14), vascular pseudo-Parkinsonism (*n =* 12), cerebellar ataxia (*n =* 4; including two cases of spinocerebellar ataxia type 6), Fahr’s syndrome (*n =* 1), normal pressure hydrocephalus (*n =* 4), and healthy participants (*n =* 2).

### Relationship between SMWI and ^123^I-FP-CIT-SPECT findings

Of the 367 participants, 293 showed loss of nigral hyperintensity on SMWI and 308 showed abnormal visual results on ^123^I-FP-CIT SPECT, with a concordance rate of 95.4% (Table 1). Among them, one participant diagnosed with idiopathic Parkinson’s disease showed loss of nigral hyperintensity on SMWI but normal finding on ^123^I-FP-CIT SPECT, presumably due to the mild decrease in striatal DaT uptake which resulted in a visually normal appearance. In addition, 16 participants showed intact nigral hyperintensity on SMWI but abnormal finding on ^123^I-FP-CIT SPECT, including idiopathic Parkinson’s disease (*n =* 12), multiple system atrophy (*n =* 1), vascular pseudo-Parkinsonism (*n =* 2), and Fahr’s syndrome (*n =* 1). The measured ^123^I-FP-CIT SBR of participants with intact nigral hyperintensity on SMWI was significantly lower than that of participants with intact nigral hyperintensity (2.36 ± 0.86 vs. 4.31 ± 1.11, *P* < 0.01).

**Table 1:** SMWI and ^123^I-FP-CIT SPECT findings in all study participants

	Intact nigral hyperintensity on SMWI (*n =* 74)	Loss of nigral hyperintensity on SMWI (*n =* 293)	*P*-value
Normal visual finding on ^123^I-FP-CIT SPECT (*n =* 59)	58/74 (78.4%)	1/293 (0.3%)	
Abnormal visual finding on ^123^I-FP-CIT SPECT (*n =* 308)	16/74 (21.6%)	292/293 (99.7%)	
			< 0.01

Values are presented as n (%)

SMWI, susceptibility map-weighted imaging; ^123^I-FP-CIT SPECT, ^123^I-2β-carbomethoxy-3β-(4-iodophenyl)-N-(3-fluoropropyl)-nortropane single-photon emission computed tomography

### Correlation of deep regressor-predicted and measured ^123^I-FP-CIT SBRs

Randomly-selected data from 293 participants (80%) were used for training the deep regressor model, and 74 participants (20%) were used for the test set (Table 2). In the test set, the predicted ^123^I-FP-CIT SBRs were significantly lower in the participants with loss of nigral hyperintensity on SMWI than in those with intact nigral hyperintensity.

**Table 2:** SMWI and ^123^I-FP-CIT SPECT findings in the test set

	Intact nigral hyperintensity on SMWI (*n =* 19)	Loss of nigral hyperintensity on SMWI (*n =* 55)	*P*-value
Normal visual finding on ^123^I-FP-CIT SPECT (*n =* 16)	16/19 (84.2%)	0/55 (0%)	
Abnormal visual finding on ^123^I-FP-CIT SPECT (*n =* 58)	3/19 (15.8%)	55/55 (100%)	< 0.01
Measured ^123^I-FP-CIT SBR	4.16 ± 1.24	2.31 ± 0.85	< 0.01
Deep regressor-predicted ^123^I-FP-CIT SBR	4.21 ± 1.35	2.44 ± 0.90	< 0.01

Values are presented as n (%) or as mean ± standard deviation

SMWI, susceptibility map-weighted imaging; ^123^I-FP-CIT SPECT, ^123^I-2β-carbomethoxy-3β-(4-iodophenyl)-N-(3-fluoropropyl)-nortropane single-photon emission computed tomography; SBR, specific binding ratio

**Fig. 2:**
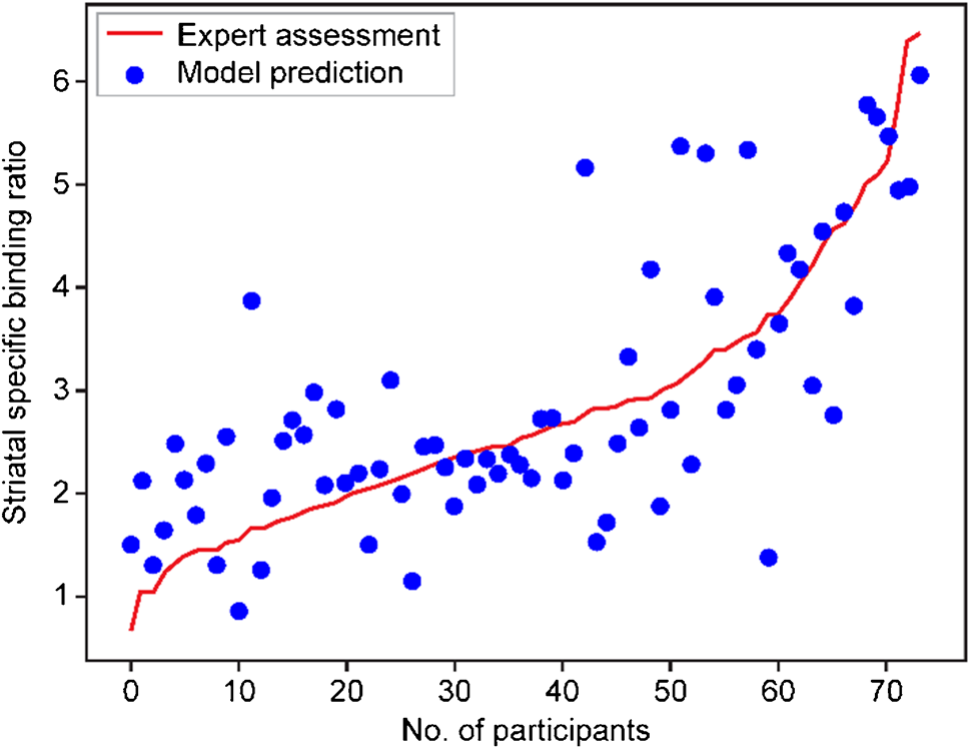
Plot of the predicted striatal SBRs against the sorted measured SBRs evaluated on ^123^I-FP-CIT SPECT. The predictions showed a good correlation of 0.744 with the SPECT evaluation. ^123^I-FP-CIT SPECT, ^123^I-2β-carbomethoxy-3β-(4-iodophenyl)-N-(3-fluoropropyl)-nortropane single-photon emission computed tomography; SBR, specific binding ratio

The sorted measured ^123^I-FP-CIT SBRs and the corresponding prediction values showed a significant positive correlation with a correlation coefficient of 0.7443 (95% CI, 0.6216–0.8314; *P* < 0.001) (Fig. 2). The average distance between the predicted and measured SBRs was 0.6837 ± 0.5972 (95% CI, 0.5467–0.8207) with a median of 0.4727. The average distance obtained for using the ResNet convolution blocks was 0.9206 ± 0.7569 (95% CI, 0.7482–1.0931), which was significantly poorer than using the proposed VGG blocks (*P* < 0.05). The root-mean-square error of the proposed regression model was 0.9078 (95% CI, 0.7183–1.0641). Considering the observed range of the measured ^123^I-FP-CIT SBR, the deep regressor showed a mean normalized error of 10.68 ± 9.39% (95% CI, 8.54%–12.82%), with a median error of 7.39%.

### Activation map

**Fig. 3:**
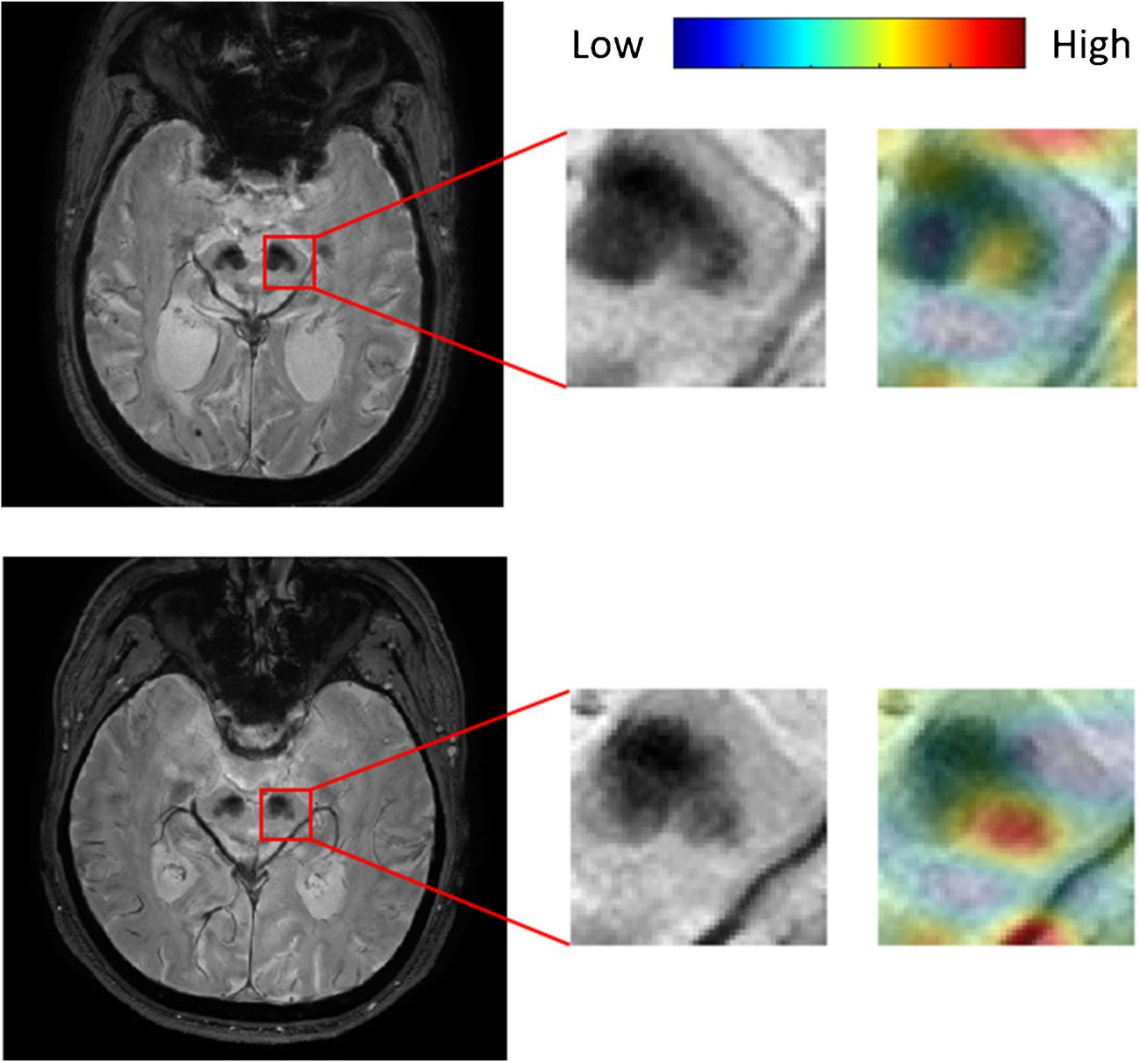
Attention map examples for low striatal^123^I-FP-CIT SBR (top) and high SBR (bottom). The leftmost images represent two examples of SMWI input. The images in the middle indicate the trimmed nigrosome patch. The images on the right show the resultant attention map blended over the input patch. The attention map is illustrated using the color map above, and an additional opacity channel, where high opacity is used for high-attention regions and low opacity is used for low-attention regions. ^123^I-FP-CIT, ^123^I-2β-carbomethoxy-3β-(4-iodophenyl)-N-(3-fluoropropyl)-nortropane; SBR, specific binding ratio

The analysis of activation maps is useful for interpreting the explicability of a deep learning model. Activation maps highlight regions of attention for prediction. Figure 3 shows the activation maps for two examples (one with low SBR and one with high SBR). High attention regions highlighting the nigrosome were observed in each of these examples.

## Discussion

In this study, we demonstrated that a deep learning regressor model based on nigrosome MRI could effectively predict striatal DaT uptake on SPECT. The results of the human-based assessment of the loss of nigral hyperintensity on 3 T SMWI and the abnormal nigrostriatal dopaminergic degeneration on SPECT were significantly correlated, and the manually measured ^123^I-FP-CIT SBRs were significantly lower with the loss of nigral hyperintensity. After training the deep-learning regressor model, it could predict ^123^I-FP-CIT SBRs based on 3 T SMWI, and the predicted values were significantly correlated with the established ground truth that was manually measured, with a correlation coefficient of 0.744. Therefore, we propose that deep learning-based analysis of non-invasive 3 T nigrosome MRI could determine the striatal DaT uptake levels in patients with Parkinsonism.

Presynaptic dopaminergic scans, such as ^123^I-FP-CIT SPECT, are more sensitive indicators of Parkinsonism than clinical examinations as they detect nigrostriatal dopaminergic degeneration [32]. DaT imaging quantifies the degree of nigrostriatal dopaminergic degeneration by measuring DaT uptake in the striatum, which is an effective tool to diagnose Parkinsonism and monitor disease progression [33]. However, current DaT imaging techniques are accompanied by ionizing radiation exposure, which makes repeated examinations in a single patient along with the disease progression clinically challenging. Its accessibility is limited compared to MRI, as the examination can be invasive due to the radiotracer injection, and its cost is relatively high.

With high-resolution MRI, the nigral structures of nigrosomes have been successfully visualized on 3 T SMWI, and their loss has been a strong diagnostic indicator of Parkinsonism. This was a breakthrough in the imaging-based diagnosis of Parkinsonism since 3 T SMWI can be applied in patients without any risk of radiation exposure and can be repeated as many times as clinically needed. Thus, the loss of nigral hyperintensity on MRI may correspond to dopaminergic denervation on DaT imaging. Striatal ^123^I-FP-CIT uptake can be significantly lower with the loss of nigral hyperintensity on SWI than with intact nigral hyperintensity [8, 14, 34, 35]. In addition, decreased striatal DaT uptake might precede the loss of nigral hyperintensity in a sequential process [14, 34]. Accordingly, 16 participants in our study showed abnormal ^123^I-FP-CIT SPECT finding but intact nigral hyperintensity on SMWI as previously reported [8, 14]. One study previously showed a large overlap between SBRs of participants with intact and non-intact nigral hyperintensities [14]. Another study also determined the quantified ^123^I-FP-CIT uptake level based on the nigral hyperintensity status on 3 T SMWI, where the nigrosome signal is lost when striatal ^123^I-FP-CIT SBRs drop below certain thresholds [34]. When nigrosomes appear morphologically normal but striatal DaT uptake slightly decreased, the MRI findings may be falsely negative for early Parkinson’s disease. Therefore, imaging-based diagnosis of Parkinsonism solely based on MRI can be misleading, and DaT imaging is necessary for a definitive diagnosis.

Nigral hyperintensity in patients with Parkinsonism may progressively decrease along with striatal DaT uptake during the disease process. However, the human eye cannot detect the partial loss of nigral hyperintensity before its total loss; thus, it can only provide a qualitative assessment, capturing either the presence or absence of nigral hyperintensity. Consequently, the demand has increased for quantifying nigral hyperintensity and determining its correlation with striatal DaT uptake for disease monitoring using SWI by adopting a deep learning-based approach. However, a recent study utilizing CNN for the deep learning-based assessment of the SN on SWI could only determine the presence or absence of nigrosome-1 but not provide any quantitative data possibly correlating with DaT imaging [36]. For the deep learning-based evaluation of the nigral SMWI prediction of SBRs, our choices for the analytic method could be either segmentation-based or regression-based estimation. The segmentation-based method can provide interpretable results if the segmentation result is visible; however, it necessitates a dedicated post-processing step, and the derived results of segmentation are a mere intermediate step to the estimation of SBRs, not an end product. However, regression-based estimation permits the direct estimation of the parameter of interest using a segmentation-free regression network, and with deep learning, it can provide more accurate end-to-end results [37]. Therefore, in our study, we chose regression-based estimation over segmentation-based estimation to predict SBRs using SMWI.

Our deep learning-based regressor model can quantitatively predict striatal DaT uptake based on 3 T SMWI in patients with Parkinsonism. The degree of decrease in striatal DaT uptake can be predicted using nigrosome MRI, making non-invasive 3 T SMWI both a diagnostic indicator and disease monitoring tool for Parkinsonism. Notably, the high attention regions on the attention maps were correlated with the regions of presenting nigral hyperintensity from the nigrosome-1; therefore, we can assume that the degree of the remaining nigral hyperintensity on SMWI was primarily used for the network to predict striatal DaT uptake. Until recently, the quantitative assessment of Parkinsonism using MRI has mainly focused on the use of neuromelanin MRI, rather than nigrosome MRI. The area and contrast ratio of T1 high-signal neuromelanin pigmentation in the SN decreased in patients with Parkinsonism [28], in correlation with striatal DaT uptake [38–40]. The area of T1 high-signal neuromelanin-rich SN is easier to segment and measure than the more irregular and smaller shape of nigral hyperintensity, leading to several deep learning-based analyses of the neuromelanin-rich SN in Parkinsonism and the prodromal phase [40–42]. Nigrosome MRI can be utilized to diagnose Parkinsonism based on the loss of nigral hyperintensity, but the quantitative correlation between nigral hyperintensity and DaT uptake cannot be performed, while the decrease in the area and the signal of the neuromelanin-rich area can be quantitatively correlated with DaT uptake but is difficult to utilize for the individual diagnosis of Parkinsonism [43]. However, as our current study has provided a quantitative analysis of SMWI-based nigrosome imaging in correlation with DaT uptake using a deep learning-based regressor model, the overall role of MRI in assessing Parkinsonism can be expanded by covering individual diagnosis, quantification, and correlation with DaT imaging, and even monitoring disease progression.

Our study had some limitations. First, as we enrolled participants with concomitant examinations of 3 T SWI and ^123^I-FP-CIT SPECT, those included were mainly diagnosed with Parkinson’s disease, whereas the number of disease control participants was unavoidably small. The number of participants in the training set was unevenly distributed among disease categories. Second, as the number of patients with isolated rapid-eye-movement sleep behavior disorder was relatively small, we could not analyze the diagnostic performance for the prodromal phase separately, and the potential strength of our regressor model in predicting phenoconversion from premotor disease could not be determined. Future studies should focus on the use of our regressor model in patients with prodromal diseases. Third, as this study was performed in a single institution, our study results could not be externally validated. It was difficult to find an external dataset that included simultaneous 3 T SMWI and ^123^I-FP-CIT SPECT images, as in our study design. Although our results demonstrate the effectiveness of a deep learning-based regressor correlating nigrosome MRI with striatal DaT imaging, further validation using external cohorts is needed. To generalize our results, variable nigrosome MRI obtained from different scanners and sites should be used for further validation. Therefore, future multicenter studies are needed to set up optimized imaging protocols for nigral MRI.

## Conclusion

Our proposed deep learning-based regressor model effectively showed that nigrosome MRI findings could be correlated with striatal DaT uptake on SPECT in the continuous domain. It could predict striatal ^123^I-FP-CIT SBRs with a high correlation with manually measured values based on 3 T SMWI. Deep learning-based regressors may help in overcoming the current limitation of nigrosome MRI and become a potential biomarker for Parkinsonism, predicting disease severity and progression.

## Supplementary information

Below is the link to the electronic supplementary material.

Supplementary file1 (PDF 184 KB)

## Data Availability

The datasets used and/or analysed during the current study are available from the corresponding author on reasonable request and after the approval of institutional ethical review board.

## Abbreviations

CI: Confidence interval
CNN: Convolutional neural network
DaT: Dopamine transporter
^123^I-FP-CIT: ^123^I-2β-carbomethoxy-3β-(4-iodophenyl)-N-(3-fluoropropyl)-nortropane
MRI: Magnetic resonance imaging
ROI: Region-of-interest
SBR: Specific binding ratio
SMWI: Susceptibility map-weighted imaging
SN: Substantia nigra
SPECT: Single-photon emission computerized tomography
SWI: Susceptibility-weighted imaging
VGG: Visual Geometry Group
3D: Three-dimensional
