# Supplementary material for: Deep learning regressor model based on nigrosome MRI in Parkinson syndrome effectively predicts striatal dopamine transporter-SPECT uptake
Source: Neuroradiology. 2023 May 20;65(7):1101–9. doi: 10.1007/s00234-023-03168-z (PMC10271910; doi:10.1007/s00234-023-03168-z)
Supplement: Supplementary file 1 — Supplementary file1 (PDF 184 KB) [file 234_2023_3168_MOESM1_ESM.pdf]

Deep learning regressor model based on nigrosome MRI in Parkinson syndrome effectively predicts striatal dopamine transporter-SPECT uptake

Yun Jung Bae[1#], Byung Se Choi[1#], Jong-Min Kim[2]*, Walid Abdullah AI[3], Ildong Yun[3], Yoo Sung Song[4], Yoonho Nam[3], Se Jin Cho[1], Jae Hyoung Kim[1]

[1]Departments of Radiology, Seoul National University Bundang Hospital, Seoul National University College of Medicine, Seongnam, Republic of Korea

[2]Departments of Neurology, Seoul National University Bundang Hospital, Seoul National University College of Medicine, Seongnam, Republic of Korea

[3]Division of Computer Engineering, Hankuk University of Foreign Studies, Yongin, Republic of Korea

[4]Departments of Nuclear Medicine, Seoul National University Bundang Hospital, Seoul National University College of Medicine, Seongnam, Republic of Korea

[#]Yun Jung Bae and Byung Se Choi contributed equally to this article

***Corresponding author**: Jong-Min Kim, MD, PhD

Department of Neurology, Seoul National University Bundang Hospital, Seoul National University College of Medicine

173-82, Gumi-ro, Bundang-gu, Seongnam-si, Gyeonggi-do 463-707, Republic of Korea

Tel.: +82-31-787-7465

Fax: +82-31-787-4059

E-mail: jongmin1@snu.ac.kr

Supplementary Materials

Methods

Technical CNN Architecture in Deep Regressor Model

The initial part of our network consisted of three convolutional blocks to extract features from the input image. The latter part uses a block of fully connected layers to predict the SBR from the extracted features. Each convolutional block had two convolutional layers followed by a max-pooling layer. Because of the 3D nigral image input, the convolutional kernels used in the convolutional layer were also 3D, with a size of $3 \times 3 \times 3$. The max-pooling layers were also 3D, with a $2 \times 2 \times 2$ pool size. A stride of 2 was used to gradually reduce the spatial dimension. The final feature maps (output of the last convolutional block) was flattened and fed into the fully connected stack for prediction.
